# Genome-Wide and Trait-Specific Markers: A Perspective in Designing Conservation Programs

**DOI:** 10.3389/fgene.2018.00389

**Published:** 2018-09-19

**Authors:** Hao Sun, Babatunde Shittu Olasege, Zhong Xu, Qingbo Zhao, Peipei Ma, Qishan Wang, Shaoxiong Lu, Yuchun Pan

**Affiliations:** ^1^Department of Animal Science, School of Agriculture and Biology, Shanghai Jiao Tong University, Shanghai, China; ^2^Shanghai Key Laboratory of Veterinary Biotechnology, Shanghai, China; ^3^College of Animal Science and Technology, Yunnan Agricultural University, Kunming, China

**Keywords:** conservation, trait-specific, SNP, QTL, population structure

## Abstract

Nowadays, breed conservation has entered the genomics era and it is imperative to develop novel theory to design the breeding schemes of the conservation populations by using the genomic information. The genome-wide markers have been regarded as a useful strategy to maintain genetic diversity. However, using the genome-wide SNPs to optimize diversity might not be optimal for some specific loci associated with specific-traits. Using the sequencing data of the conserved population of the Saba pig breed, we demonstrated that the conservation program designed by using the genome-wide SNPs might result in the loss of the genetic diversity of the reproduction trait. We suggested an idea of using phylogenetic tree to select valuable individuals for conservation program based on the genome-wide and trait-specific makers. The selection rule was to make the selected samples to be widely distributed as much as possible in both the genome-wide and trait-specific phylogenetic trees.

The genetic diversity of the indigenous pig breeds guarantees the sustainable development of pork industry. However, due to the excellent production performance of the Western commercial pig breeds (Ai et al., 2013), they have dominated the pig industry, thereby leading to a dramatic reduction in the population size of the indigenous pig breeds (Fang et al., 2005; Kim et al., 2005). China is one of the leading countries in terms of genetic resources for domestic pigs, having more than one hundred indigenous breeds (Ai et al., 2015). Most of these breeds are renowned for excellent performance in reproduction, meat quality and adaptation (Bosse et al., 2014). Therefore, many state-owned conservation farms have been set up for raising those indigenous pig breeds in China.

In the past, the conservation programs were designed based on the pedigree information. Nowadays, the development of high-throughput genotyping techniques has made it possible to obtain a large amount of genomic markers in pig breeds. By using these markers, pedigree reconstruction could correct pedigree errors and recover hidden relatedness (Klapste et al., 2017). The genome-wide information has been regarded as a useful strategy to maintain genetic diversity (de Cara et al., 2011; Bosse et al., 2015). However, the conservation program designed by the genome-wide SNPs might result in the loss of the genetic diversity of some special traits, and thus, result in reduced performance. In general, the indigenous pig breeds have their special performance traits. These traits are important genetic resource and maintaining the genetic diversity means maintaining the phenotype variation. Since the sample size of conservation population is usually small, gene drift may occur easily and the alleles that contribute to the special traits might also be lost. Therefore, it is crucial to maintain the genetic diversity of these special traits. We suggested an approach of using phylogenetic tree to select valuable individuals for conservation program based on the genome-wide and trait-specific makers. Here, we describe the details of our simple framework by using the sequencing data of the conservation population of the Saba pig breed.

A total of 108 distantly related Saba pigs (males: 30; females: 78) from the state-owned conservation farm were selected. By using the GGRS (genotyping by genome reducing and sequencing) protocol (Chen et al., 2013), a total of 211 654 high-confidence SNPs with minor allele frequencies (MAFs) ≥ 0.05 were obtained. These SNPs were widely distributed in the genome (**Figure 1**). By using all the SNPs, a pairwise distance matrix (1-IBS) was obtained by Plink v1.07 (Purcell et al., 2007). Based on the pairwise distance matrix, the phylogenetic tree was constructed using the neighbor-joining method (**Figure 2A**) (Saitou and Nei, 1987). The phylogenetic tree can directly reflect the genetic distance among pigs which can foster easy selection of individuals for breeders. Since Saba pig breed is renowned for its good performance in reproduction, we set the SNPs associated with the reproduction trait as the trait-specific markers. The pig QTL database^1^ was used to identify the candidate genome area related to the reproduction traits. QTL terms with large span (up to 246 Mb, QTL_ID = 5223) were filtered. Empirically, we set the threshold distance to 1 Mb. A total of 17 814 SNPs associated with the reproduction trait were identified, and a phylogenetic tree was also constructed by using these SNPs. (**Figure 2B**).

**FIGURE 1:**
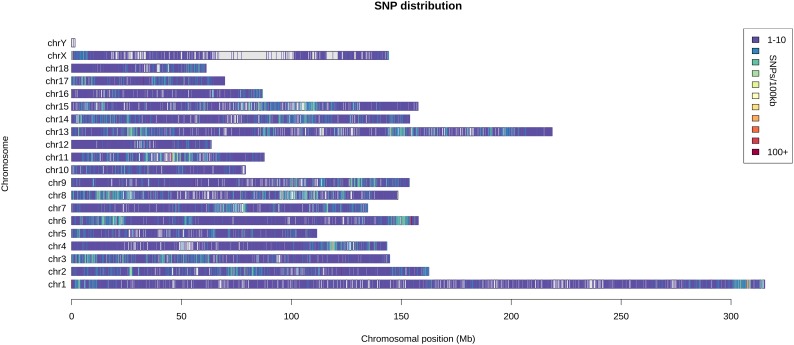
The distribution of the SNPs in the chromosomes.

**FIGURE 2:**
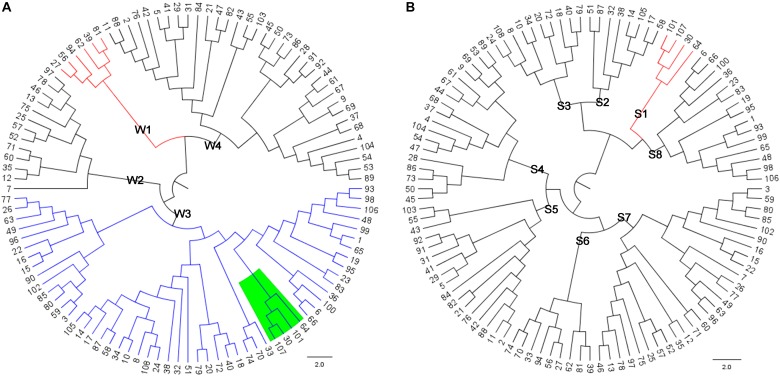
**(A)** Phylogenetic tree constructed by using the genome-wide SNPs; **(B)** Phylogenetic tree constructed by using the SNPs associated with reproduction trait.

According to phylogenetic trees, we assumed that there are four pedigrees in **Figure 2A** and eight pedigrees in **Figure 2B**. Assuming that the conservation program was constructed only by the genome-wide SNPs, the individuals 30, 64, 101, 107 (green shade in **Figure 2A**, red color lines in **Figure 2B**) might not be selected as the breeding pigs, and thus, the genetic diversity will be lost for the reproduction traits. If we aimed to select pigs for conservation as well as maintain the genetic diversity of reproduction traits, we should select samples that both represent the four pedigrees in **Figure 2A** and eight pedigrees in **Figure 2B**. According this rule, we can then design our conservation program.

For example, assuming that we need to select 16 pigs from the Saba pig population, we should select two pigs from each pedigree in **Figure 2B** and at the same time ensures that the selected 16 pigs represent the four pedigrees in **Figure 2A**. The pigs 39 and 56 can be selected to both represent the pedigrees W1 and S6. The pigs 7 and 75 can be selected to represent the pedigrees W2 and S7. The pigs 30, 107, 14, 51, 18, 108, 1, 23 can be selected to represent the pedigrees W3 and S1, S2, S3, S8. The pigs 86, 73, 2, and 43 can be selected for represent the pedigrees W4 and S4, S5. Therefore, the 16 pigs from the four pedigrees in **Figure 2A** and eight pedigrees in **Figure 2B** can be properly represented. Hence, the conservation program based on this illustrated framework can maintain the genetic diversity in both genome-wide and trait specific level.

Nowadays, breed conservation has entered the genomics era. Breeders tend to use the genomic markers to reconstruct pedigree in order to reveal the relationship among animals and select valuable individuals. Some indigenous pig breeds have their own special characters (traits) and it is important to preserve these traits. Our concern is that the utilization of all markers across the genome to optimize genetic diversity might not be optimal for other specific loci associated with specific-traits. Therefore, we suggested that the combination of genome-wide and trait-specific markers could be crucial in designing conservation programs.

We postulated a simple framework to illustrate how the idea can be implemented in practice. Simply, the QTL database was used to identify the trait-specific SNPs. The relationships among samples were explored based on the (1-IBS) distance by using genomic-wide and trait-specific markers. The two phylogenetic trees based on the relationship matrix were used to select valuable individuals by ensuring that they are widely distributed as much as possible in both the genome-wide and trait-specific trees. To obtain the trait-specific markers, we used the QTL database since it can provide us with many candidate regions for specific traits. However, combining the QTL results across previous studies can be very challenging because these studies differ in many aspects such as markers, breeds, and statistical methods.

Overall, we suggested that the conservation programs should be designed by combining the information of genome-wide and trait-specific markers. Moreover, this conservation program is not only suitable for the indigenous pig breeds but might also be adopted for the conservation of genetic resource of other farm animals such as cattle, goat, sheep, chicken etc.

## Ethics Statement

Animals were treated according to institutional guidelines, and the study protocol was approved by the Research Committee of Shanghai Jiao Tong University.

## Author Contributions

HS, YP, and QW conceived the study. SL collected the samples. HS, BO, ZX, QZ, and PM helped to performed the experiments and analyses of the study. HS wrote the manuscript. All authors have read and edited the manuscript.

## Conflict of Interest Statement

The authors declare that the research was conducted in the absence of any commercial or financial relationships that could be construed as a potential conflict of interest.

## Footnotes

1 https://www.animalgenome.org/cgi-bin/QTLdb/SS/index

## References

[B1] Ai H. S. Fang X. D. Yang B. Huang Z. Y. Chen H. Mao L. K. (2015). Adaptation and possible ancient interspecies introgression in pigs identified by whole-genome sequencing. *Nat. Genet.* 47 217–225. 10.1038/ng.3199 25621459

[B2] Ai H. S. Huang L. S. Ren J. (2013). Genetic diversity, linkage disequilibrium and selection signatures in chinese and western pigs revealed by genome-wide SNP markers. *PLoS One* 8:e56001. 10.1371/journal.pone.0056001 23409110 PMC3567019

[B3] Bosse M. Megens H. J. Frantz L. A. F. Madsen O. Larson G. Paudel Y. (2014). Genomic analysis reveals selection for Asian genes in European pigs following human-mediated introgression. *Nat. Commun.* 5:4392. 10.1038/Ncomms5392 25025832 PMC4225517

[B4] Bosse M. Megens H. J. Madsen O. Crooijmans R. P. M. A. Ryder O. A. Austerlitz F. (2015). Using genome-wide measures of coancestry to maintain diversity and fitness in endangered and domestic pig populations. *Genome Res.* 25 970–981. 10.1101/gr.187039.114 26063737 PMC4484394

[B5] Chen Q. Ma Y. F. Yang Y. M. Chen Z. L. Liao R. R. Xie X. X. (2013). Genotyping by genome reducing and sequencing for outbred animals. *PLoS One* 8:e67500. 10.1371/journal.pone.0067500 23874423 PMC3715491

[B6] de Cara M. A. Fernandez J. Toro M. A. Villanueva B. (2011). Using genome-wide information to minimize the loss of diversity in conservation programmes. *J. Anim. Breed. Genet.* 128 456–464. 10.1111/j.1439-0388.2011.00971.x 22059579

[B7] Fang M. Hu X. Jiang T. Braunschweig M. Hu L. Du Z. (2005). The phylogeny of Chinese indigenous pig breeds inferred from microsatellite markers. *Anim. Genet.* 36 7–13. 10.1111/j.1365-2052.2004.01234.x 15670125

[B8] Kim T. H. Kim K. S. Choi B. H. Yoon D. H. Jang G. W. Lee K. T. (2005). Genetic structure of pig breeds Korea from analysis China using microsatellite loci. *J. Anim. Sci.* 83 2255–2263. 10.2527/2005.83102255x 16160034

[B9] Klapste J. Suontama M. Telfer E. Graham N. Low C. Stovold T. (2017). Exploration of genetic architecture through sib-ship reconstruction in advanced breeding population of *Eucalyptus nitens*. *PLoS One* 12:e0185137. 10.1371/Journal.Pone.0185137 28938023 PMC5609769

[B10] Purcell S. Neale B. Todd-Brown K. Thomas L. Ferreira M. A. R. Bender D. (2007). PLINK: a tool set for whole-genome association and population-based linkage analyses. *Am. J. Hum. Genet.* 81 559–575. 10.1086/519795 17701901 PMC1950838

[B11] Saitou N. Nei M. (1987). The neighbor-joining method: a new method for reconstructing phylogenetic trees. *Mol. Biol. Evol.* 4 406–425. 10.1093/oxfordjournals.molbev.a040454 3447015

